# Unveiling Endogenous Serum Peptides as Potential Biomarkers for Hepatocellular Carcinoma in Patients with Liver Cirrhosis

**DOI:** 10.1021/acs.jproteome.4c00269

**Published:** 2024-08-23

**Authors:** Muhammad Salman Sajid, Yuansong Ding, Rency S. Varghese, Alexander Kroemer, Habtom W. Ressom

**Affiliations:** † Department of Oncology, Lombardi Comprehensive Cancer Center, Georgetown University Medical Center, Washington, D.C. 20057, United States; ‡ MedStar Georgetown Transplant Institute, MedStar Georgetown University Hospital and the Center for Translational Transplant Medicine, Georgetown University Medical Center, Washington, D.C. 20057, United States

**Keywords:** liver cancer, endogenous peptides, biomarker, serum, nano-LC-MS/MS

## Abstract

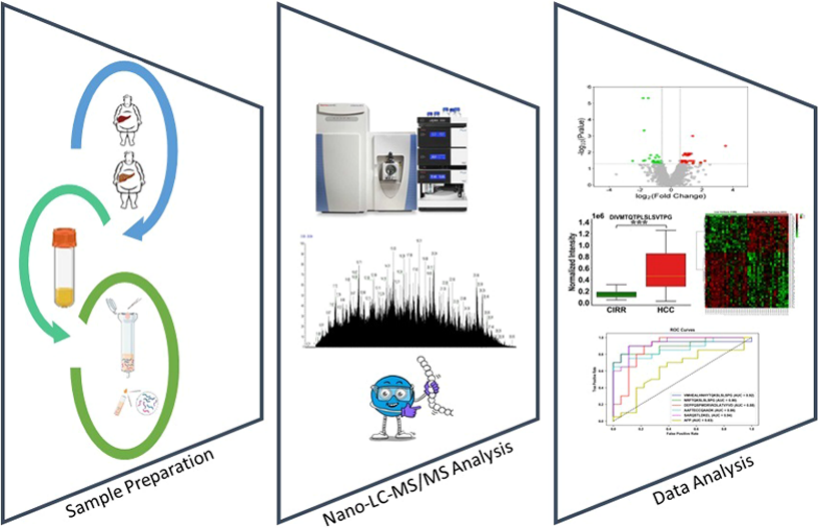

Hepatocellular carcinoma (HCC) is a leading cause of cancer-related deaths worldwide, mainly associated with liver cirrhosis. Current diagnostic methods for HCC have limited sensitivity and specificity, highlighting the need for improved early detection and intervention. In this study, we used a comprehensive approach involving endogenous peptidome along with bioinformatics analysis to identify and evaluate potential biomarkers for HCC. Serum samples from 40 subjects, comprising 20 HCC cases and 20 patients with liver cirrhosis (CIRR), were analyzed. Among 2568 endogenous peptides, 67 showed significant differential expression between the HCC vs CIRR. Further analysis revealed three endogenous peptides (VMHEALHNHYTQKSLSLSPG, NRFTQKSLSLSPG, and SARQSTLDKEL) that showed far better performance compared to AFP in terms of area under the receiver operating characteristic curve (AUC), showcasing their potential as biomarkers for HCC. Additionally, endogenous peptide IAVEWESNGQPENNYKT that belongs to the precursor protein Immunoglobulin heavy constant gamma 4 was detected in 100% of the HCC group and completely absent in the CIRR group, suggesting a promising diagnostic biomarker. Gene ontology and pathway analysis revealed the potential involvement of these dysregulated peptides in HCC. These findings provide valuable insights into the molecular basis of HCC and may contribute to the development of improved diagnostic methods and therapeutic targets for HCC.

## Introduction

Hepatocellular carcinoma (HCC) is the most prevalent liver cancer and the second leading cause of cancer-related deaths globally.^1^ It is predicted that the annual number of new liver cancer cases will exceed one million by 2025. This projection suggests that HCC will emerge as a leading cause of cancer mortality in many developed countries, including the United States.^2^ Liver resection and transplantation are currently the most effective treatments for HCC with the potential for cure. Unfortunately, these treatment options are hampered by limited availability and can only be utilized during the early stages of the disease.^3^ Since it is often diagnosed at advanced stages, as symptoms are typically absent in the early stages, HCC is a particularly aggressive and difficult-to-treat cancer. Several protein biomarkers like alpha-fetoprotein (AFP), Alpha-l fucosidase (AFU), Glypican-3 (GCP-3), Golgi protein-73 (GP-73), Squamous cell carcinoma antigen (SCCA), des-gamma-carboxyprothrombin (DCP), β2 microglobulin, osteopontin and squalene epoxidase, etc. have been identified as potential indicators for early detection of HCC.^4^

Alpha fetoprotein is commonly used as a serologic biomarker for HCC with inadequate sensitivity (40–64%). Additionally, elevated AFP levels are observed in only 20% of HCC patients.^5^ Furthermore, it has been observed that cirrhotic or HCC patients may exhibit abnormal AFP levels.^6^^7^ Patients with liver cirrhosis have a higher risk of developing HCC, as approximately 90% of HCC cases are linked to long-standing cirrhosis.^8^ The progression of cirrhosis to HCC is often fatal due to the lack of reliable biomarkers for early diagnosis during the later stages of HCC development. Discovering potent biomarkers for the early detection of HCC in patients with liver cirrhosis is crucial for improving patient outcomes. These biomarkers help revolutionize the management of cirrhosis, enabling early intervention and personalized treatment approaches. Moreover, they can serve as valuable tools for clinical trials, allowing for better selection of patients and assessment of the treatment efficacy.

The use of human blood for diagnostic analyses in clinical practice is well-established due to its easy accessibility and abundance of disease-related proteins and peptides.^9^ While serum/plasma proteomics has been extensively studied in recent times, there has been relatively less research on the low-molecular-weight (LMW) plasma/serum endogenous peptides.^10^ Endogenous peptides are small chains of amino acids that are naturally produced within the body and reflect the protease activity in body fluids and tissues.^11^ They play important roles in diverse physiological processes such as metabolism, and immune response, and peptide fragments that are produced through proteolytic processes within the tissue microenvironment may serve as indicators of early stage pathophysiological alterations.^12^ Due to their involvement in multiple biological pathways and stability in biological samples, it is believed that these peptides could serve as valuable biomarkers for disease detection, monitoring, and treatment response. Endogenous peptide study in the blood is difficult due to their low abundance. Therefore, it is important to develop effective methods for extracting and detecting these peptides.^13^ Several techniques, such as nuclear magnetic resonance (NMR) spectroscopy,^14^ Circular dichroism Spectroscopy,^15^ Fourier-transform infrared spectroscopy (FT-IR),^16^ and X-ray crystallography,^17^ have been employed by researchers to gain a deeper understanding of their function and potential therapeutic applications. Currently, mass spectrometry (MS) remains an indispensable analytical tool in proteomics and other omics studies^18^ due to its accurate measurement of peptide masses, elucidation of peptide sequences, the profile of the peptidome in a high-throughput manner, and study of post-translational modifications. These features enable researchers to unravel the complex world of peptides and their roles in biological systems. Liquid chromatography (LC) coupled with tandem mass spectrometry (MS) is an important technique for studying endogenous peptides due to its high sensitivity and specificity. LC-MS allows for the separation, identification, and quantification of peptides in complex biological samples, providing valuable insights into their abundance, sequence, and modifications at very low concentrations that have been used for biomarker discovery.^19^

In this research article, we investigate the low-molecular-weight proteome, also known as the endogenous peptidome, in serum samples from patients with liver cirrhosis (CIRR) and HCC. The goal is to identify potential biomarkers that could lead to more accurate detection of HCC in the high-risk population of patients with liver cirrhosis.

## Materials and Methods

### Experimental Design and Specimen Collection

The study cohort consisted of 40 subjects (20 cases of HCC and 20 patients with CIRR). All subjects were recruited from MedStar Georgetown University Hospital (MGUH) with the approval of the Georgetown University Institutional Review Board (IRB). Informed consent forms and Health Insurance Portability and Accountability Act (HIPAA) authorization forms were obtained from all participants. HCC cases were diagnosed using established diagnostic imaging criteria and histology, and clinical stages were determined using the tumor-node-metastasis (TNM) staging system. The two patient groups (HCC and CIRR) did not differ significantly in terms of gender, age, or other clinical traits. Table 1 provides the detailed characteristics of the subjects. For blood collection, peripheral venipuncture was performed using sterile vacuum tubes without anticoagulants. The blood was centrifuged at room temperature, first at 1000 × g (10 min) followed by another 10 min at 2500 × g. The resulting serum supernatant was carefully collected, divided into aliquots with protease inhibitor, and stored at −80 °C until further analysis.

**Table 1:** Demographic and Clinical Data for Sample Set (n = 40)

	Serum	HCC (*N* = 20)	CIRR (*N* = 20)	p-Value
Age	*Mean (SD)*	59 (6)	57 (6)	0.2859
Gender	*Male*	12	13	1.0000
Race	*AA*	10	8	0.3300
	*EA*	10	12	
BMI	*Mean (SD)*	30 (7.2)	29.5 (5.1)	0.7964
Etiology	*HCV*	17	16	1.0000
	*Alcohol*	6	6	1.0000
HCV Serology	*HCV Ab+*	16	15	0.6948
HBV Serology	*anti HBC+*	9	8	0.7431
	*HBs Ag+*	1	0	1.0000
Smoking	*Current*	5	5	1.0000
	*Former*	11	10	
	*None*	4	5	
Alcohol	*Current*	5	4	1.0000
	*Former*	11	12	
	*None*	4	4	
AFP	*Median (IQR)*	29.1 (60.8)	7 (35.1)	0.3101
AST	*Median (IQR)*	107.5 (83.2)	97 (73)	0.8425
ALT	*Median (IQR)*	98.5 (53.2)	58 (50.5)	0.0655
MELD	*Median (IQR)*	10.5 (5.2)	14.5 (10)	0.0359
Child Pugh score	*Mean (SD)*	6.8 (1.8)	9.1 (2.8)	0.0116
	*Median (IQR)*	6 (2.5)	9 (5)	
Child-Pugh Class	*A*	9	2	
	*B*	7	9	
	*C*	3	6	
HCC Stage	*Stage I*	6	None	
	*Stage II*	13		
	*Stage III*	1		

### Sample Preparation

A modified version of Wang et al.’s^20^ protocol was used to extract endogenous peptides from human serum samples. Briefly, 40 μL of serum was mixed with 250 μL of 1% trifluoroacetic acid (TFA) and vortexed for 30 s. The mixture was then heated at 98 °C for 10 min to disrupt peptide–protein interactions. After cooling, the samples were transferred into an Amicon Ultra-0.5 centrifugal filter unit with a molecular weight cutoff (MWCO) of 10 kDa. Prior to transfer, the filter unit was preconditioned with 150 μL of 70% ethanol with 1% TFA. The samples were centrifuged at 14,000 × g for 20 min at 4 °C, washed twice with 100 μL of 1% TFA, and centrifuged for additional 10 min. The extracted endogenous peptides were then transferred into new sample vials and subjected to freeze-drying before the subsequent desalting steps.

The released endogenous peptides were purified using BioPureSPN Mini, PROTO 300 C18 columns (The Nest Group, Inc., MA, USA). To prepare the columns for desalting, 250 μL of 50% ACN was used for equilibration, followed by conditioning with 250 μL of 2% TFA. The dried peptides were suspended in 100 μL of 2% TFA and loaded onto the column, repeated once. The column was then washed twice with 100 μL of 2% TFA. The captured peptides were eluted using 100 μL of 80% ACN, 1% TFA, followed by 100 μL of 50% ACN, 1% TFA. All centrifugation steps were performed using an Eppendorf benchtop centrifuge at 100 × g for 1 min. Finally, the purified endogenous peptides were dried and resuspended in 2% ACN, 0.1% FA before quantification and injection into nano-LC-MS/MS.

### Nano-LC-MS/MS Analysis

Positive mode MS data were collected using a Dionex 3000 UltiMate Nano LC system coupled to a Q-Exactive mass spectrometer (Thermo Scientific, San Jose, CA, USA). A 1 μg portion of endogenous peptides was injected into the LC system for analysis. To ensure optimal purification and separation, samples were first passed through a C18 Acclaim PepMap trap column (75 μm × 20 mm, 3 μm, 100 Å, Thermo Scientific) before being transferred to a C18 Acclaim PepMap RSLC column (75 μm × 250 mm, 3 μm, 100 Å, Thermo Scientific). A multistage gradient was used for the chromatographic separation, with a total run time of 145 min. The mobile phase A was water and 0.1% formic acid (FA), while mobile phase B was 80% acetonitrile (ACN) and 0.1% FA. The column oven temperature was maintained at 35 °C throughout the run. During the initial 5 min, the mobile phase B was held constant at 4% with a flow rate of 300 nL/min. It was then gradually increased to 35% over the next 120 min with a flow rate of 220 nL/min. Between 125 and 133 min, mobile phase B was further increased to 95% with a flow rate of 250 nL/min, and this composition was maintained for 5 min. Finally, the mobile phase B was reduced to 4% with a flow rate of 300 nL/min and held constant until the end of the run. The separated endogenous peptides were directed to the Q-Exactive mass spectrometer using a nanospray flex ion source at a voltage of 2.2 kV. The full MS scan was performed with a scan range of 370–1850 *m*/*z*, and the analytes were detected in the Orbitrap at a resolution of 70000. The top 10 most intense ions were selected for MS2 fragmentation in a high-energy collision dissociation (HCD) cell, with a normalized collision energy (NCE) of 27.5 at a resolution of 17500, with a dynamic exclusion of 30 ms.

### Mass Spectrometry Data Processing

Peptide identification and label-free quantification (LFQ) were performed using Proteome Discoverer (PD) 3.0 software (Thermo Scientific, USA). The search was conducted against the Uniprot database of human proteins (July 2022) using the Sequest HT search algorithm. The processing workflow involved the use of the mass recalibration node, Minora Feature Detector, standard spectrum selector, Sequest HT, and Percolator nodes. To address the presence of chimeric spectra, a precursor detector node was employed. Precursor mass tolerance was set at 10 ppm, and fragment mass tolerance was set to 0.02 Da with no specific enzyme being used. Static modifications were not considered, but variable peptide modifications, such as oxidized methionine, were included. False discovery rate (FDR) tolerances in the Percolator node were set at 0.01 for high confidence and 0.05 for medium confidence.

### Data and Bioinformatics Analysis

Clustering within each sample group was assessed by principal component analysis (PCA) using LFQ intensities of all peptides to determine potential outliers. Peptides that were detected in less than 70% of at least one sample group were filtered out. The abundance data for all filtered peptides were log2-transformed, and missing values inherent to DDA shotgun approaches were imputed using the KNN featurewise method. The data were then normalized by median to reduce variation between runs. To identify statistically significant peptides between the HCC and CIRR groups, a Student’s *t* test was performed. Differentially expressed peptides with fold change (FC) above 1.5 and FDR < 0.05 were identified. The peptides were used by the Ingenuity Pathway Analysis (IPA) software for pathway and functional correlation analysis. To determine the proportion of specific peptides, present or absent, we categorized the leftover peptides after filtering out those detected by more than 70% of the samples in each sample group. Pearson’s chi-squared test was used to assess if there were any significant differences in the presence or absence of each peptide between the two groups at each time point.

## Results and Discussion

**Figure 1:**
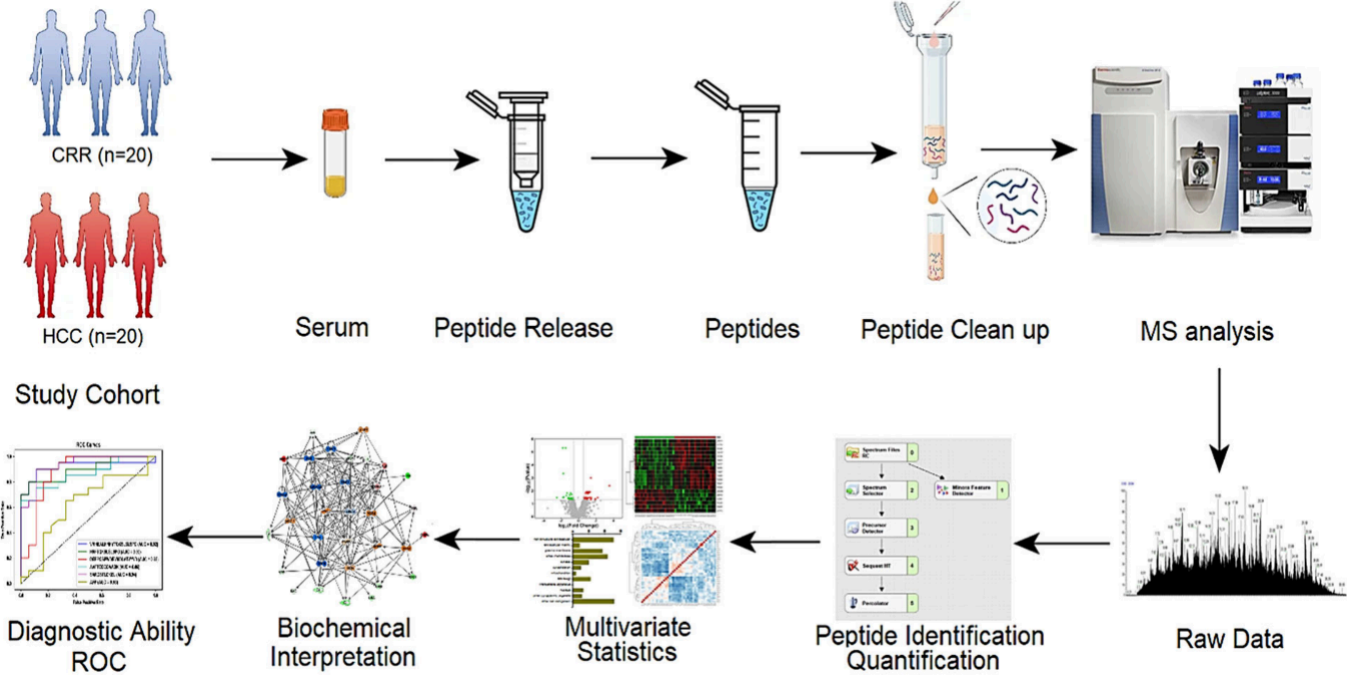
Workflow of nano-LC-MS/MS-based endogenous peptide analysis for HCC biomarker discovery.

Endogenous peptides, naturally occurring peptides with 3 to 100 amino acids, are widely distributed across cells, tissues, and body fluids. Mainly these can be generated by precursor protein degradation (e.g., by peptidases/protease), direct gene encoding, and gene-independent enzymatic formation. The analysis of endogenous peptides has garnered significant attention due to their importance in health and disease.^21^ Recent evidence indicates endogenous peptides hold great potential as a source of clinically relevant cancer biomarkers due to their stability, personalized nature, and ability to shed light on cancer biology.^22^ This study aimed to identify new potential endogenous peptide biomarkers for HCC by using label-free mass spectrometry on serum samples from patients with liver cirrhosis. The overall strategy and simplified workflow are shown in Figure 1. A total of 40 serum samples, 20 HCC cases, and 20 patients with liver cirrhosis were analyzed. To evaluate instrument reproducibility, we loaded a standardized reference sample, the HeLa protein digest standard from Pierce, with a quantity of 200 ng, nine times between the serum samples. The intensity distribution for the HeLa protein digest standard showed a minimal variation. The average coefficient of variation (CV) for 2327 proteins detected across nine runs was less than 16%. In addition, we evaluated the correlation among the 40 serum samples based on all identified endogenous peptides whose median intensity ranged from 4 × 10^–6^ to 3 × 10^–9^. The average Pearson correlation coefficient among these samples was 0.9 (Figure S1), demonstrating that the LC-MS platform showed an acceptable performance.

**Figure 2:**
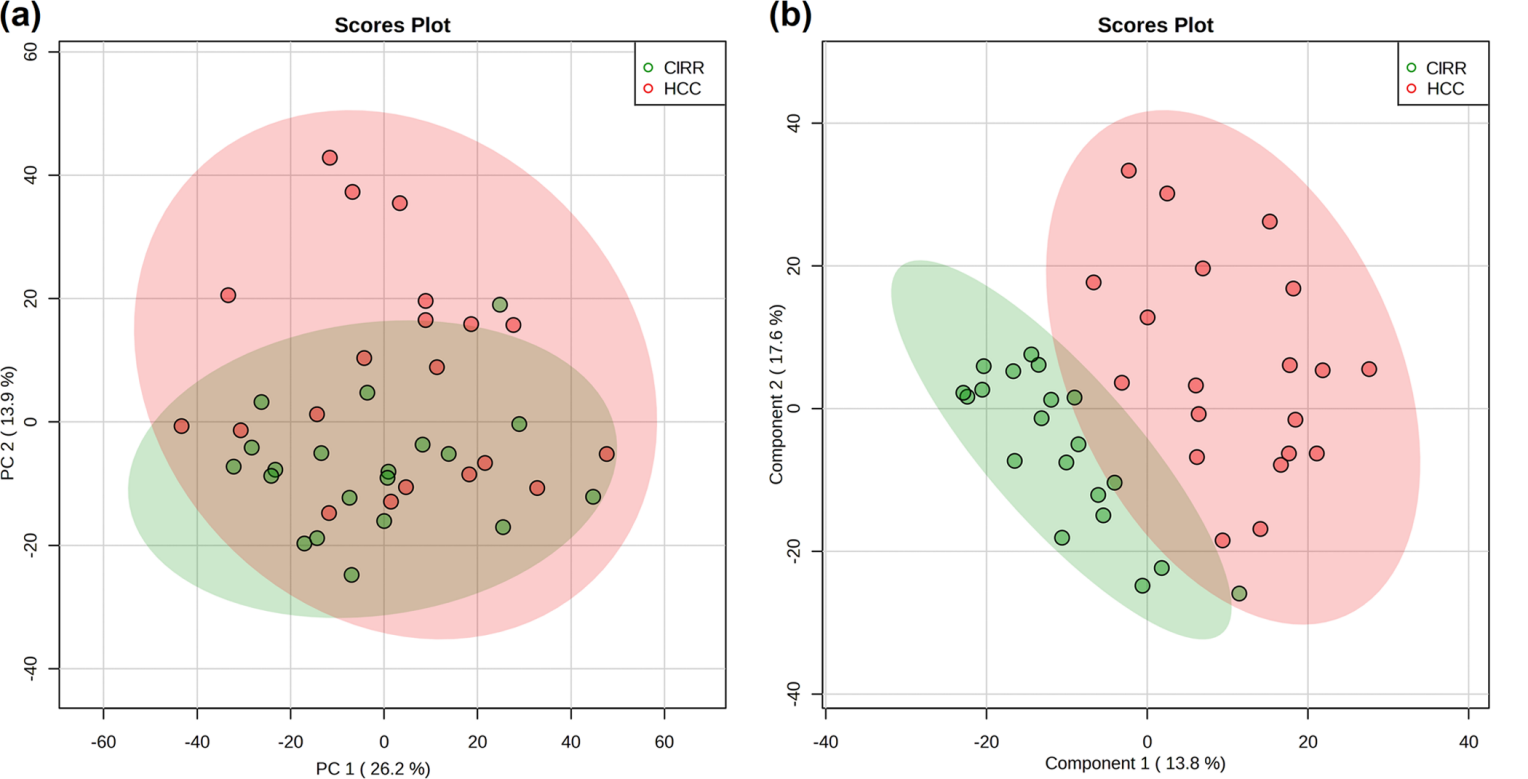
Score plots of PCA (a) and PLS-DA (b) depicting HCC (red) and CIRR (green) cohorts.

Endogenous peptide data generated from nano-LC-Q-Exactive-MS were subjected to PCA and PLS-DA analyses to visualize the differences between HCC and CIRR. The PCA score plot revealed some overlap between the HCC and CIRR groups (Figure 2a), which could be due to the similar disease states in these cohorts. The PLS-DA score plot in Figure 2b shows good modeling and prediction capabilities, with R^2^ = 0.99 and Q^2^ = 0.575. High values of R^2^ and Q^2^ closer to 1 indicate an excellent model with high explanation and prediction capacities.^23^ The overall R^2^ and Q^2^ values indicate that the model has good predictability, suggesting a distinct differentiation between HCC and CIRR groups.

The nano-LC-MS/MS analysis detected a total of 2568 endogenous peptides. These peptides were identified through 857,525 spectra (PSM), with sequences ranging from 7 to 43 amino acids (Figure S2). They were generated by the cleavage of 269 precursor proteins; the details of all the identified peptides are provided in Table S1. In the CIRR group, 2118 unique peptides were identified. In the HCC group, 2241 unique peptides were identified. Some MS/MS spectra of peptides that are uniquely identified in HCC but not in the CIRR group and vice versa are shown in Figure S8 and S9. Upon comparison of the two cohorts, it was found that 1791 peptides were common between the CIRR and HCC groups, as illustrated by the Venn Diagram (Figure S3). Out of the 269 precursor proteins, 205 were found in the CIRR group and 241 in the HCC group. Of these, 177 precursor proteins were common in both groups, as illustrated by the Venn Diagram (Figure S4).

**Figure 3:**
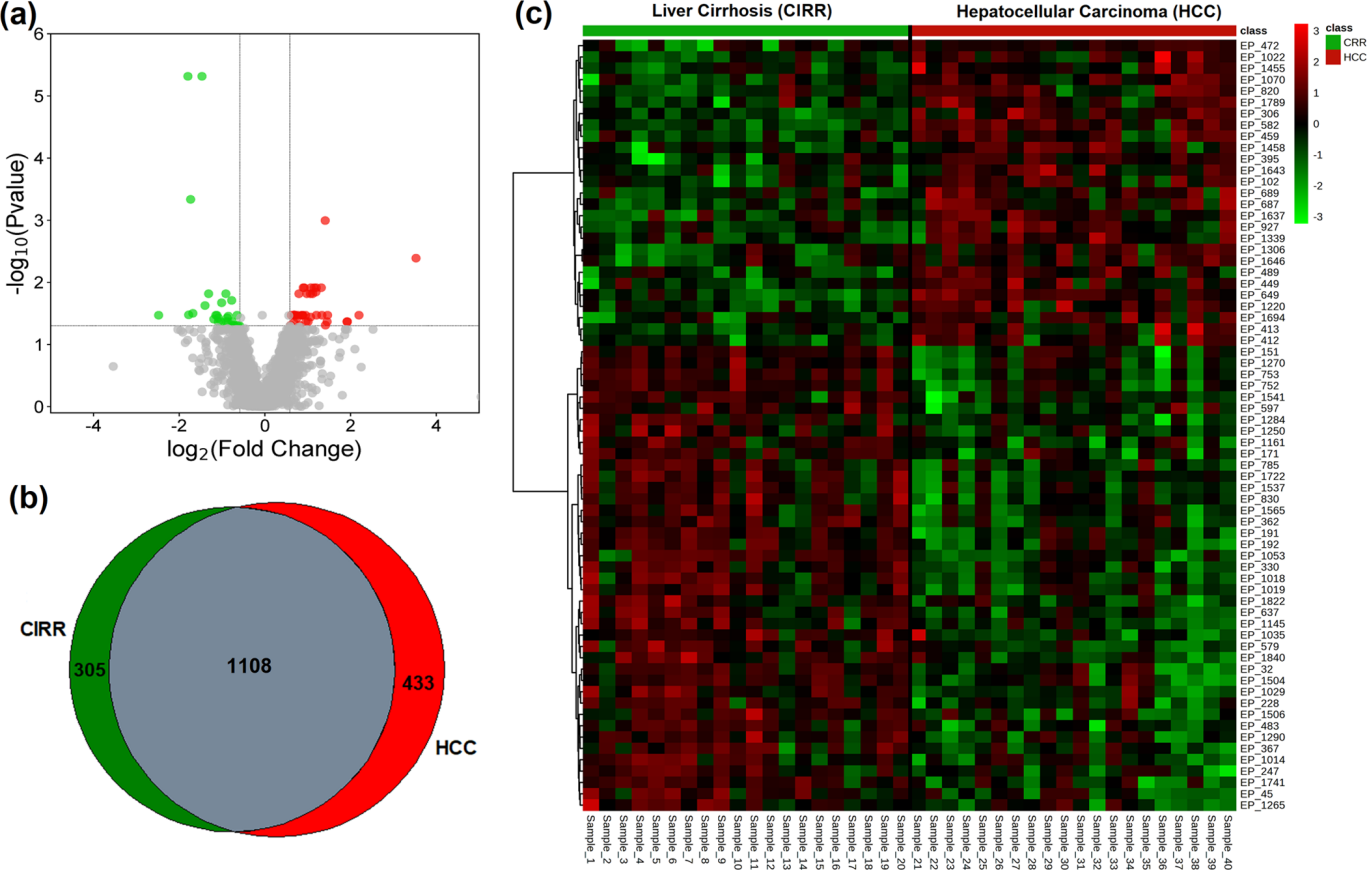
(a) Volcano plot highlighting significantly up- and down-regulated endogenous peptides with |FC| > 1.5 and FDR < 0.05. (b) Venn diagram of 1903 endogenous peptides detected in more than 70% samples in each group (CIRR and HCC). (c) Heatmap of serum endogenous peptides differently expressed in HCC vs CIRR.

To compare the peptidomes of HCC patients with those of CIRR, we used a label-free quantification approach to quantify the peptides in the serum samples. We filtered the data by considering only peptides that were present in at least 70% of the samples in each group. Out of the 2568 peptide sequences that were identified were found, 1846 were based on this criterion, and 1108 peptides appeared commonly in both groups (Figure 3b). The details of these filtered peptides are provided in Table S2. We calculated the fold change by comparing the average abundance of the target peptide in HCC versus CIRR cohorts. A 1.5-fold change, with an FDR less than 0.05, was considered as the cutoff for determining differential expression. In addition, we selected peptides as potential marker candidates based on their detectability in the majority of patients and their signal-to-noise ratio for mass spectrometry-based quantitation. Among all the filtered peptides, we observed that 67 showed a fold change over 1.5 and FDR ≤ 0.05, indicating a significant alteration between the two groups. Figure 3c depicts a heatmap of these 67 endogenous peptides, 29 up-regulated and 38 downregulated in HCC vs CIRR. As illustrated in Table 2, these peptides originated from 31 precursor proteins. Figure S5 depicts a heatmap of all identified endogenous peptides.

**Table 2:** List of Significantly Dysregulated Serum Peptides in HCC vs CIRR

Precursor Proteins	Sequence	Charge	*m*/*z* [Da]	RT [min]	p-Value	FDR	Fold Change
Histone H4	TVTAMDVVYALK	2	663.8539	92.33	0.0007692	0.033753	5.58
Immunoglobulin heavy constant gamma 2	VMHEALHNHYTQKSLSLSPG	3	755.7086	50.83	5.2316 × 10^–09^	0.00000483	3.47
	SVMHEALHNHYTQKSLSLSPG	3	784.7179	53.70	0.0002007	0.019507	1.71
	PIEKTISKTKGQPRE	3	571.3247	20.17	0.00071075	0.033753	2.32
Immunoglobulin kappa variable1-5	DIQMTQSPSTLSASVGDR	2	954.9544	69.23	0.00043325	0.033324	3.43
Keratin, type I cytoskeletal 18	TVQSLEIDLDSMR	2	761.8778	87.20	0.0003753	0.031496	3.20
Immunoglobulin heavy constant gamma 3	NRFTQKSLSLSPG	3	478.9296	57.74	7.502 × 10^–07^	0.0004617	2.97
C4b-binding protein alpha chain	SARQSTLDKEL	3	416.5602	35.84	4.0083 × 10^–09^	0.00000483	2.77
Albumin	AAFTECCQAADK	2	686.2883	38.88	0.0002682	0.023576	2.63
Alpha-1-antiproteinase	SVLGDVGITEVFSDR	2	797.4104	120.04	0.0006232	0.015221	2.48
Immunoglobulin kappa constant	TVAAPSVFIFPPSDEQLKSG	2	1045.5439	115.76	0.0010807	0.039118	2.29
	STYSLSSTLTLSKAD	2	787.4022	89.53	0.0017085	0.048894	1.67
	DSTYSLSSTLTLSKAD	2	844.9140	99.17	0.0018882	0.049794	1.65
Immunoglobulin kappa variable 1D-8	DIVMTQTPLSLSVTPG	2	837.9356	119.05	0.0005386	0.033753	2.21
	EIVMTQSPATLSVSP	2	788.4064	91.76	0.0018605	0.049794	1.62
Alpha-2-macroglobulin	SVSGKPQYMVLVPSLLH	3	624.3429	94.28	0.0007192	0.033753	2.17
Fibrinogen alpha chain	DSGEGDFLAEGGG	2	605.7507	89.32	0.00097818	0.037229	2.12
	DSGEGDFLAEGG	2	577.2392	90.06	0.0012801	0.042894	2.12
	SSSYSKQFTSS	2	604.7758	36.05	0.00099226	0.037229	1.84
Apolipoprotein A-I	LATVYVDVLKDSG	2	690.3772	95.80	0.0014173	0.042894	2.03
Immunoglobulin kappa variable 1-33	DIQMTQSPSSLSASVGDRVT	2	1047.9998	80.17	0.00023059	0.021284	2.01
Complement C3	GVFQEDAPVIHQEMIG	2	893.4300	90.27	0.00011589	0.015221	1.88
Immunoglobulin kappa variable 3-11	EIVLTQSPATLSLSPG	2	806.9465	117.43	0.0012223	0.042894	1.87
	EIVLTQSPATLSLSPGE	2	871.4658	118.17	0.00085114	0.034915	1.80
Alpha-1-antitrypsin	TEEAKKQINDYVEK	3	565.6236	35.56	0.0013021	0.042894	1.82
Immunoglobulin heavy constant gamma 4	ALTSGVHTFPAVL	2	656.8663	97.31	0.0012449	0.042894	1.70
	NSGALTSGVHTFPAVLQSSG	2	965.4929	95.89	0.001681	0.048894	1.62
Immunoglobulin gamma-1 heavy chain	TLVTVSSASTKGPSVF	2	790.9320	86.19	0.0007511	0.033753	1.61
	GTLVTVSSASTKGPSVFPLAPSSK	3	773.4270	90.26	0.001874	0.049794	1.50
Apolipoprotein A-I	DEPPQSPWDRVKDLATVYVD	2	1165.5688	133.58	0.00001104	0.0040773	–11.3
	RHFWQQDEPPQSPWDRVKD	3	817.7252	80.08	0.00060445	0.033753	–2.34
Immunoglobulin gamma-1 heavy chain	PEVKFNWYVDGVEVHNAKTKPREEQY	5	633.3206	78.20	0.00062322	0.033753	–4.25
	PIEKTISKAKGQPR	4	388.9837	19.25	0.00173	0.048894	–1.70
Fibrinogen alpha chain	ADEAGSEADHEGTHSTKRGHAKSRPV	5	546.8611	17.87	0.0014174	0.042894	–3.61
	MADEAGSEADHEGTHSTKRGHAKSRPV	4	716.0844	23.42	0.001411	0.042894	–3.51
	SYKMADEAGSEADHEGTHSTKRGHAKSRPV	6	543.4249	24.44	0.0013833	0.042894	–2.62
	GHKEVTKEVVTSED	3	519.9304	25.07	0.00089891	0.036074	–2.01
Kininogen-1	KGRPPKAGAEPASEREVS	4	467.2482	18.03	0.0017604	0.048894	–2.76
	GRPPKAGAEPASEREVS	3	579.9696	21.55	0.00009291	0.014294	–2.29
	RPPKAGAEPASEREVS	3	560.9585	21.05	0.00004538	0.012184	–2.12
Immunoglobulin lambda-1 light chain	GSPVKAGVETTKPSKQSNNK	3	686.3671	15.47	0.00052326	0.033753	–2.58
	SPVKAGVETTKPSKQ	3	519.6259	19.04	2.1942 × 10^–06^	0.0010126	–2.55
	SPVKAGVETTKPSKQSN	3	586.6507	18.97	0.00011315	0.015221	–2.01
	PVKAGVETTKPSK	3	447.9313	20.45	0.00059176	0.033753	–1.61
	GSPVKAGVETTKPSKQ	3	538.6330	20.79	0.00064197	0.033753	–1.54
Serotransferrin	VKHQTVPQNTGGKNPD	3	573.9605	16.63	0.00005254	0.012184	–2.39
Tensin-4	QVEAKATCFLPSPG	2	752.8883	108.09	0.00006956	0.012184	–2.30
Alpha-1B-glycoprotein	AIFYETQPSLWAESE	2	885.9177	131.64	0.00013205	0.015221	–2.21
Immunoglobulin heavy constant alpha 1	SGKSAVQGPPERD	2	664.3333	19.07	0.0013128	0.042894	–2.05
Immunoglobulin lambda constant 2	GVETTTPSKQSNNKYAA	3	599.2993	28.02	0.0014995	0.044646	–1.32
Apolipoprotein B-100	GTLASKTKGTFAHRD	4	398.2154	25.55	0.0013736	0.042894	–1.99
Alpha-1-antitrypsin	AAQKTDTSHHDQDHPTFN	3	683.9740	21.28	0.00006517	0.012184	–2.17
	MGKVVNPTQK	2	551.3066	22.24	0.00059935	0.033753	–1.97
	PQGDAAQKTDTSHHDQDHP	3	695.6344	15.48	0.00007260	0.012184	–1.89
	PQGDAAQKTDTSHHDQDHPT	3	729.3158	15.51	0.00005964	0.012184	–1.88
	GKVVNPTQK	2	485.7895	67.88	0.00051521	0.033753	–1.8
	KTDTSHHD	2	470.7067	19.26	0.00042204	0.033324	–1.64
	AAQKTDTSHHDQDHPTF	3	645.9574	22.88	0.00066829	0.033753	–1.57
	PQGDAAQKTDTSHHDQ	3	579.2591	19.35	0.0007134	0.033753	–1.51
	EDPQGDAAQKTDTSHHDQDH	3	744.6430	18.92	0.0017746	0.048894	–1.49
Alpha-2-macroglobulin	PKGNRIAQWQSFQLEG	3	620.3247	87.67	0.00056593	0.033753	–1.84
	PKGNRIAQWQSFQLE	3	601.3158	87.86	0.00099823	0.037229	–1.64
	RQLNYKHYD	3	412.8750	24.35	0.00013752	0.015221	–1.72
Complement C2	GNDHSLWRVNVGD	2	734.8512	71.93	0.00078622	0.033753	–1.78
Alpha-2-antiplasmin	LKLVPPMEEDYPQFGSPK	3	692.3565	98.97	0.00076583	0.033753	–1.78
Ceruloplasmin	NIKTYSDHPEKVNKD	3	596.6331	22.14	0.00014266	0.015221	–1.90
Gelsolin	GTGQKQIWRIEGSNKVPVD	3	704.7131	63.79	0.00058625	0.033753	–1.57

The upregulated peptides are from precursor proteins including Apolipoprotein A-I (2 peptides), Apolipoprotein A-I (3 peptides), Immunoglobulin heavy constant gamma 2 (2 peptides), Immunoglobulin heavy constant gamma 4 (2 peptides), Immunoglobulin kappa constant (3 peptides), and Immunoglobulin kappa variable 3-11 (2 peptides). The levels of these proteins are reported to be higher in HCC cases than in the normal controls.^24,25^ The downregulated peptides are from precursor proteins including Alpha-1-antitrypsin (9 peptides), Alpha-2-macroglobulin (3 peptides), Fibrinogen alpha chain (4 peptides), Immunoglobulin gamma-1 heavy-chain (2 peptides), Immunoglobulin lambda-1 light chain (5 peptides), Isoform LMW of Kininogen-1 (3 peptides), Serotransferrin (1 peptide), and Tensin-4 (1 peptide). The levels of these proteins are reported higher in HCC cases vs normal controls.^26^^–31^ This opposite trend may be due to two reasons (i) difference in the study cohort, i.e. our study uses CIRR as a control instead of normal controls, and the production of these proteins may be less in patients with CIRR; and (ii) decrease of protease activity of these proteins may result in low production of peptides.

**Figure 4:**
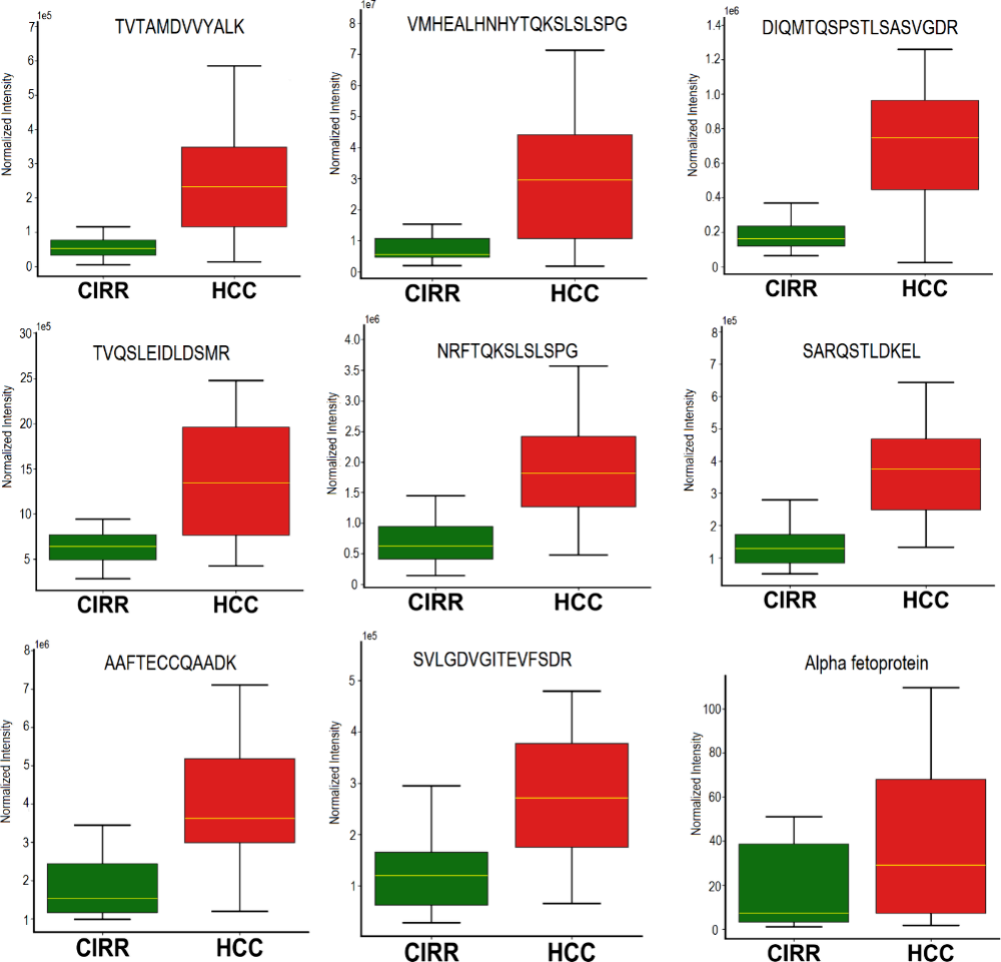
Box plots for eight endogenous peptides upregulated in HCC vs CIRR compared to alpha-fetoprotein (AFP) measured in the clinic having p-value <0.0001.

**Figure 5:**
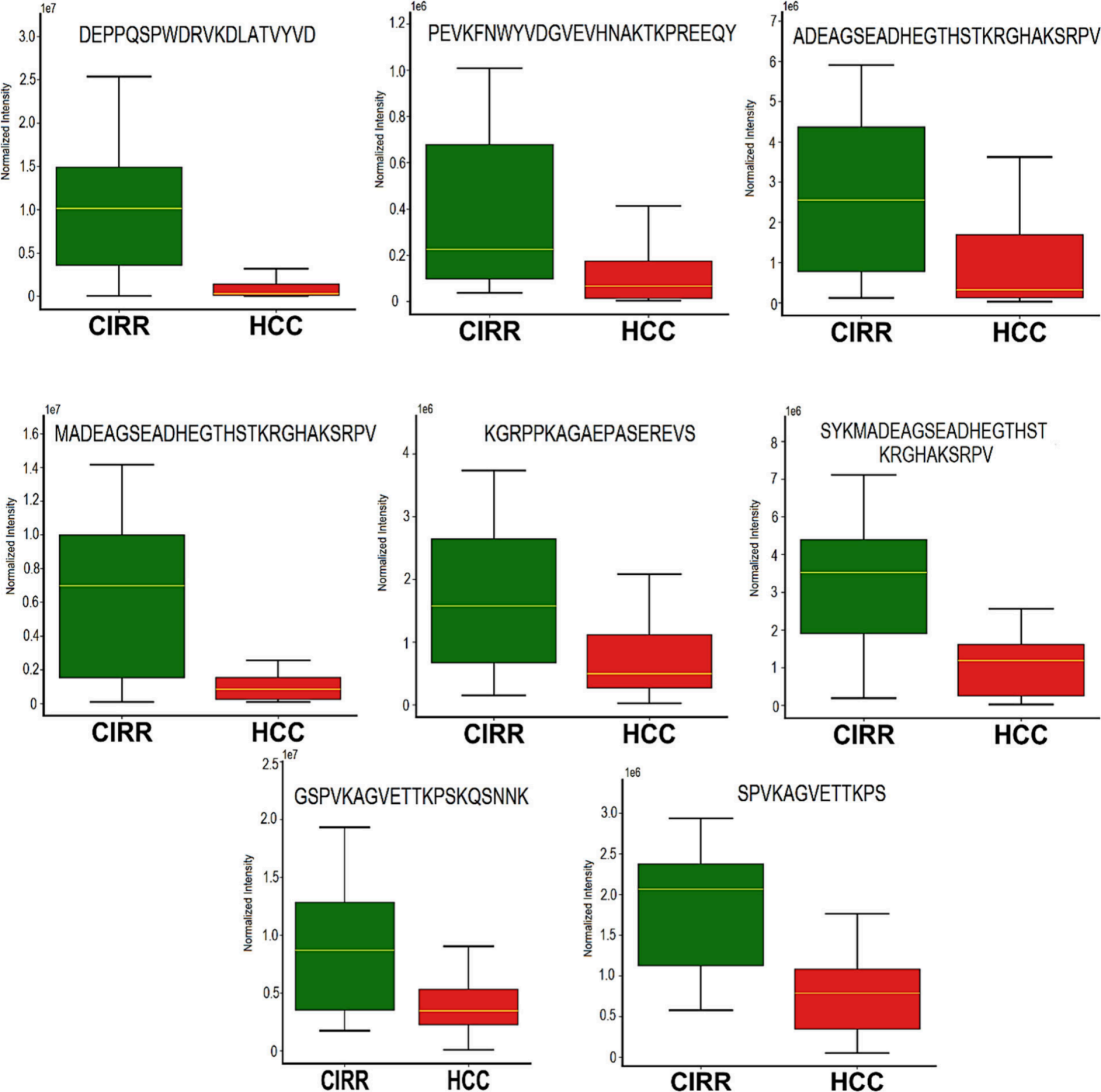
Box plots for eight endogenous peptides downregulated in HCC vs CIRR having p-value <0.0001.

Figure 4 depicts box plots of the following top eight endogenous peptides that are significantly increased in HCC vs CIRR with FDR < 0.05: TVTAMDVVYALK (5.58 FC), VMHEALHNHYTQKSLSLSPG (3.47 FC), DIQMTQSPSTLSASVGDR (3.43 FC), TVQSLEIDLDSMR (3.20 FC), NRFTQKSLSLSPG (2.97 FC), SARQSTLDKEL (2.77 FC), AAFTECCQAADK (2.63 FC), SVLGDVGITEVFSDR (2.48 FC). Also, a box plot for AFP measured by ELISA in the clinic is presented in Figure 4 for comparison. Box plots of the rest of the elevated endogenous peptides are provided in Figure S6. Similarly, Figure 5 presents box plots for the following top eight endogenous peptides that are significantly decreased in HCC vs CIRR: DEPPQSPWDRVKDLATVYVD (−11.36, FC), PEVKFNWYVDGVEVHNA-KTKPREEQY (−4.25, FC), ADEAGSEADHEGTHSTKRGH-AKSRPV (−3.61, FC), MADEAGSEADHEGTHSTKRGHAKSRPV (−3.51, FC), KGRPPKAGAEPASEREVS (−2.7 FC). SYKMADEAGSEADHEGTHSTKRGHAKSRPV (−2.62, FC), GSPVKAGVETTKPSKQS-NNK (−2.58, FC), SPVKAGVETTKPSKQ (−2.58, FC). Box plots of the rest of the downregulated peptides are provided in Figure S5.

### Diagnostic Performance

**Figure 6:**
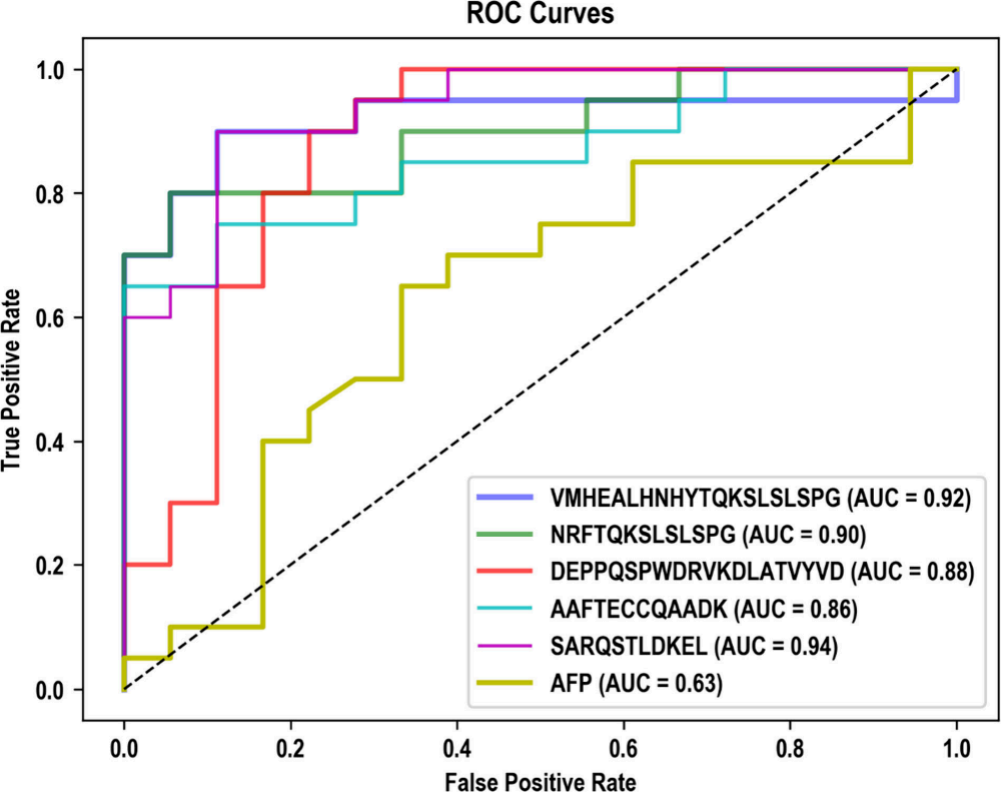
ROC curves of the top five endogenous peptides having the highest AUC values in comparison with those of AFP.

In this study, we compared the performance of several potential biomarker candidates to AFP. We found that these candidates outperformed AFP, as demonstrated by their AUC values. We found 68 differently expressed endogenous peptides that yielded AUC values between 0.72 and 0.92, whereas the AUC for AFP was 0.63. Figure 6 depicts the Receiver Operating Characteristic (ROC) curves for the top five endogenous peptides as well as AFP. While all five including AAFTECCQAADK (P02768) and DEPPQSPWDRVKDLATVYVD (P02647) showed better performance than AFP, VMHEALHNHYTQKSLSLSPG (P01859), NRFTQKSLSLSPG (P01860), and SARQSTLDKEL (P04003) yielded far better performance compared to AFP. Expression levels of our biomarker candidates in HCC stages I, II, and III vs CIRR are shown in Figure S8. SVMHEALHNHYTQKSLSLSPG, SARQSTLDKEL, AAFTECCQAADK, and NRFTQKSL-SLSPG show increased expression in stages I, II, and III compared to CIRR while DEPPQSPWDRVKDLATVYVD showed an inverse trend. Furthermore, expression levels of these markers are notably higher in stages II and III compared to stage I, which could be attributed to changes in these proteases’ activity or expression levels due to the disease state. This suggests the potential of candidates as biomarkers for HCC in the high-risk population of patients with liver cirrhosis. This is of course a preliminary finding over a limited sample size, and a larger cohort of patients is needed to confirm our findings.

The presence versus absence of individual peptides was assessed by applying the chi-square test for 224 endogenous peptides detected in less than 70% of samples in each of the two groups. The details are provided in Table S3. We focused only on those endogenous peptides that were present over 75% in one group and completely absent in another group. We found 14 endogenous peptides that met this criterion. The details of these peptides and their precursor proteins are provided in Table S4. For example, IAVEWESNGQPENNYKT that belongs to the precursor protein Immunoglobulin heavy constant gamma 4 (P01861) and LFMGKVVNPTQK that belongs to precursor protein Alpha-1-antitrypsin (P01009) were detected in 100% and 95% of the HCC samples, respectively, but they were not detected in any of the samples from the CIRR group. Similarly, RPSGIPERFSGSNSGNTATLTISRVEAGDEAD belonging to precursor protein Immunoglobulin lambda variable 3-21 (P80748) and GGKYAATSQVLLPSKDVMQGTD belonging to precursor protein Immunoglobulin heavy constant mu (P01871) were detected in 95% and 85% of samples of the CIRR group, respectively, but they were not detected in any of the samples from the HCC group. These four endogenous peptides selected on the basis of presence and absence are promising biomarker candidates for HCC.

### Molecular Pathway Analysis

To investigate the functional biological aspects of the 31 precursor proteins that were statistically significant between HCC and CIRR, we utilized Ingenuity Pathway Analysis (IPA). This analysis aimed to identify canonical pathways and potential regulatory networks and predict upstream regulators and causal relationships associated with these proteins. Figure 6a shows 15 canonical pathways with the highest statistical significance (p-value of ≤10^–3^). The liver X receptor and retinoid acid X receptor (LXR/RXR) pathways were found to be the most statistically significant pathways. These pathways are involved in regulating cholesterol and fatty acid metabolism. Out of the 31 precursor proteins analyzed, 16 are associated with this pathway, including A1BG, ALB, APOA1, APOB, C3, FGA, KNG1, SERPINA1, SERPINF2, and TF. Other highly enriched pathways included the FXR/RXR pathway, Acute Phase Response Signaling, DHCR24 Signaling Pathway, DHCR24 Signaling Pathway, Post-translational protein phosphorylation, Binding and Uptake of Ligands by Scavenger Receptors, Regulation of Insulin-like Growth Factor (IGF) transport and uptake by IGFBPs, Response to elevated platelet cytosolic Ca2+, PI3K Signaling in B Lymphocytes, and B Cell Development.

**Figure 7:**
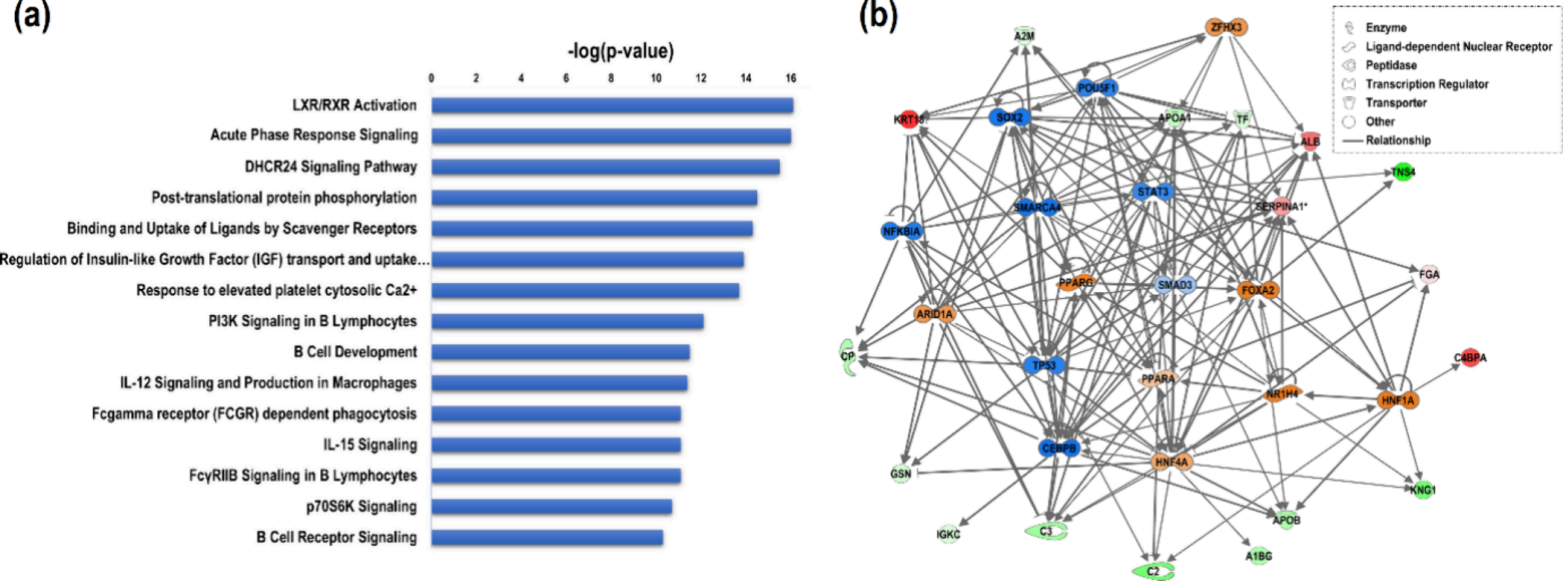
(a) Canonical pathways associated with the precursor proteins listed in Table 2. The horizontal bars indicate the negative logarithm function of the overlap p-value, which represents the statistical significance of the pathway’s association with the specified proteins. (b) A network of the 15 upstream regulator molecules significantly associated with the genes encoding the proteins listed in Table 2.

Finally, we analyzed the differentially expressed precursor proteins to identify the top 15 upstream regulators that were statistically significantly associated. We used a p-value cutoff of ≤10^–3^ and found a variety of regulators, including transmembrane receptors, ligand-dependent nuclear receptors, and enzymes. This information is presented in Figure 7b and in detail in Table S5. Hepatocyte nuclear factor 1α (HNF1α), hepatocyte nuclear factor 4α (HNF4α), and Keratin 18 (KRT18) are three transcription factors that are highly expressed in hepatocytes and play important roles in regulating liver-specific genes. They are known to act as regulators of gene expression in the liver. Unbalanced HNF1α expression is linked to both HCC and CIRR.^27^ SREBF1 plays a role in producing cholesterol and lipids by controlling the activity of around 30 relevant genes.^28^ KRT18 is involved in the regulation of cell proliferation and apoptosis genes.^29^Figure 6b represents the upstream regulator network, depicted as a graph that illustrates the molecular association among these proteins. The significant precursor proteins listed in Table 2 are also identified in this graph. Table S4 depicts the result of a causal network analysis performed by using IPA. These findings serve as only preliminary evidence for functional interactions and should be considered as hypothesis-generating, at best. To draw more definitive conclusions, further experimental validation is necessary.

## Concluding Remarks

In this research article, a low-molecular-weight serum endogenous proteome is analyzed to identify potential biomarkers that could lead to more accurate detection of HCC in the high-risk population of patients with liver cirrhosis. Among the significant differential expression endogenous peptides, we identified five potential candidates that outperform AFP in terms of their AUC values. However, to validate these findings and establish their clinical utility, large cohort studies with independent validation are needed. Our future work will focus on targeted quantitation and validation of the identified candidates in an independent cohort with a larger number of participants.

## Data Availability

Data generated in this work is available via the ProtemeXchange Consortium via the PRIDE partner repository with the identifier PXD051029.

## Supporting Information Available

Figure S1: Correlation among serum samples. Figure S2: Histogram of peptide sequence length for the serum endogenous peptides detected. Figure S3: Endogenous peptides overlapping between HCC and CIRR. Figure S4: Precursor proteins overlapping between HCC and CIRR. Figure S5: Heatmap of all serum endogenous peptides detected in HCC and CIRR. Figure S6: Box plots of significantly up-regulated serum endogenous peptides. Figure S7: Box plots of significantly down-regulated serum endogenous peptides. Figure S8: Candidate expressions in HCC stages I, II, III, and CIRR. Figure S9: MS/MS spectrum of peptides found in HCC only. Figure S10: MS/MS spectrum of peptides found in CIRR only. Table S4: Details of endogenous peptides present in one group and absent in another group. Table S5: Top 15 upstream regulators. (PDF)
Table S1: Details of 2,568 endogenous peptides identified. Table S2: Details of endogenous peptides after filtration and imputation. Table S3: Results of Chi-Square test. Table S6: Results of Causal Network Analysis using IPA. (XLSX)

## Notes

Serum samples were collected from subjects recruited at MedStar Georgetown University Hospital (MGUH) with the Georgetown University Institutional Review Board (IRB) approval.
