# Supplementary material for: Unveiling Endogenous Serum Peptides as Potential Biomarkers for Hepatocellular Carcinoma in Patients with Liver Cirrhosis
Source: J Proteome Res. 2024 Aug 23;23(9):3974–83. doi: 10.1021/acs.jproteome.4c00269 (PMC11385380; doi:10.1021/acs.jproteome.4c00269)
Supplement: Supplementary file 1 — pr4c00269_si_001.pdf [file pr4c00269_si_001.pdf]

Unveiling Endogenous Serum Peptides as Potential Biomarkers for Hepatocellular Carcinoma in Patients with Liver Cirrhosis

Muhammad Salman Sajid[1], Yuansong Ding[1], Rency S. Varghese[1], Alexander Kroemer[2], Habtom W. Ressom[1*]

[1] Department of Oncology, Lombardi Comprehensive Cancer Center, Georgetown University Medical Center, Washington, DC 20057, USA.

[2] MedStar Georgetown Transplant Institute, MedStar Georgetown University Hospital and the Center for Translational Transplant Medicine, Georgetown University Medical Center, Washington, DC, 20057, USA.

*Corresponding Author
Prof. Habtom W. Ressom
Department of Oncology
Genomics and Epigenomics Shared Resource
Lombardi Comprehensive Cancer Center
Georgetown University Medical Center
Washington, DC 20057, USA
Email: hwr@georgetown.edu

Table of Content

Table S1: Details of 2,568 endogenous peptides identified (XLSX)

Table S2: Details of endogenous peptides after filtration and imputation (XLSX)

Table S3: Results of Chi-Square test (XLSX)

Figure S1: Correlation among serum samples 3

Figure S2: Histogram of peptide sequence length for the serum endogenous peptides detected 3

Figure S3: Endogenous peptides overlapping between HCC and CIRR 4

Figure S4: Precursor proteins overlapping between HCC and CIRR 4

Figure S5: Heatmap of all serum endogenous peptides detected in HCC and CIRR groups 5

Figure S6: Box plots of significantly up-regulated serum endogenous peptides 6

Figure S7: Box plots of significantly down-regulated serum endogenous peptides 7

Figure S8: Candidate expressions in HCC stages I, II, III, and CIRR. 8

Figure S9: MS/MS spectrum of peptides found in HCC only 9

Figure S10: MS/MS spectrum of peptides found in CIRR only 12

Table S4: Details of endogenous peptides present in one group and absent in another group 13

Table S5: Top fifteen upstream regulators 14

Table S6: Results of Causal Network Analysis using IPA (XLSX)

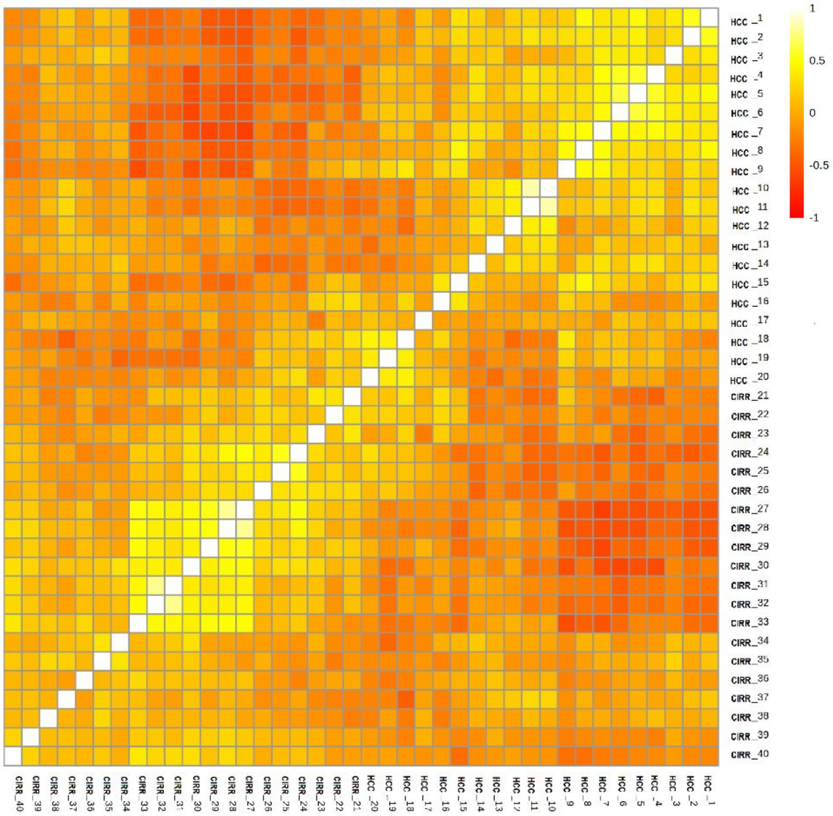

Figure S1: Correlation among the serum endogenous peptide profiles from 40 subjects.

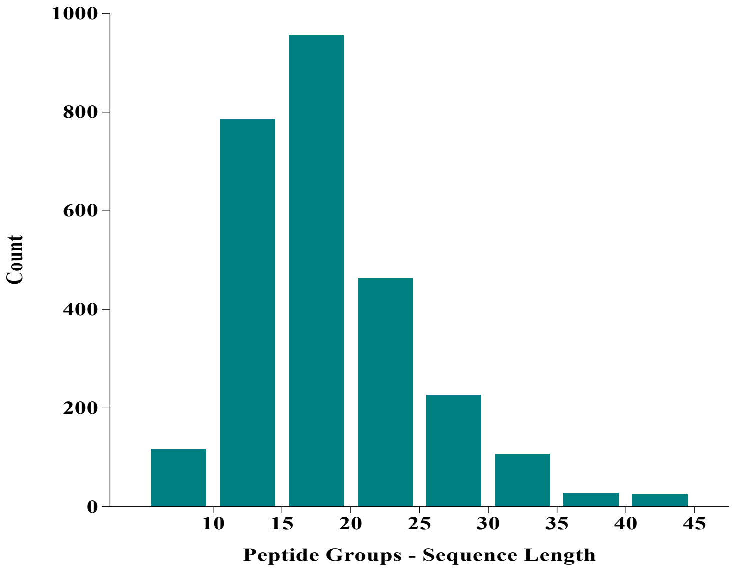

Figure S2: Histogram of peptide sequence length for the serum endogenous peptides detected in the 40 samples.

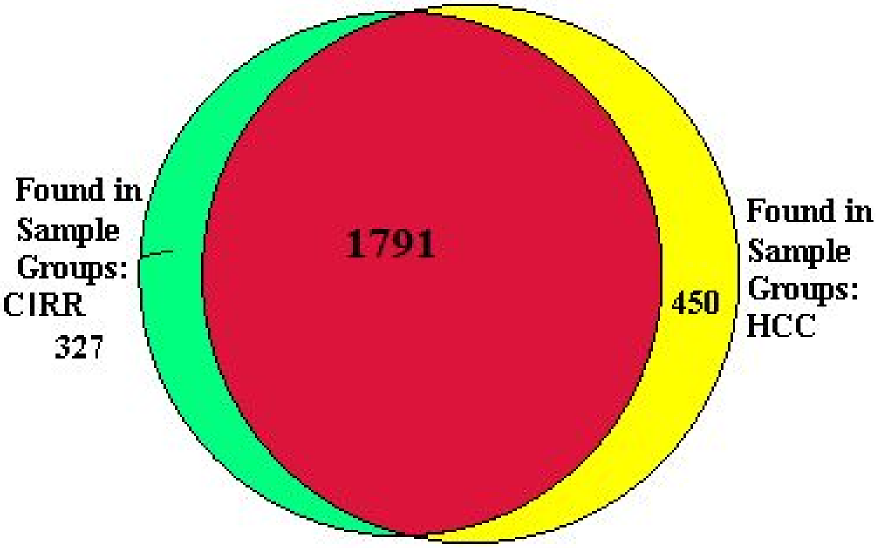

Figure S3: Venn diagram of the number of peptides detected in HCC vs. CIRR.

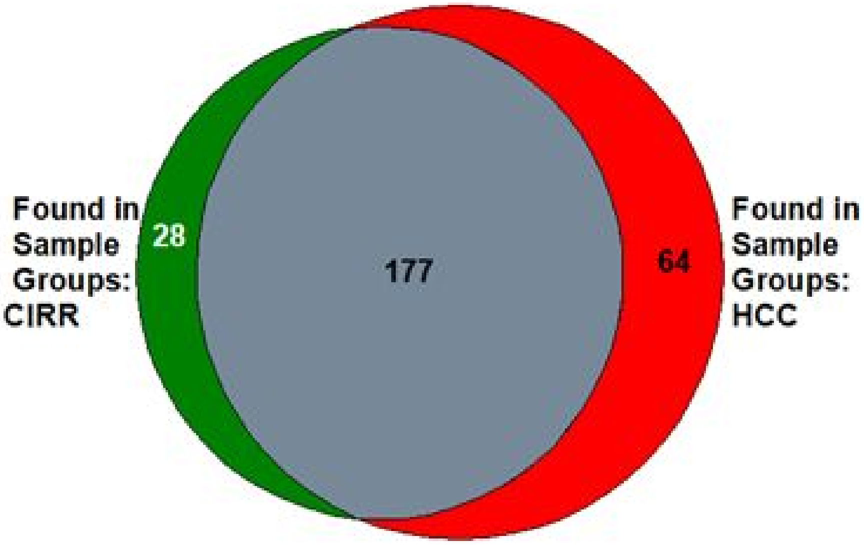

Figure S4: Venn diagram of the number of proteins identified in HCC vs. CIRR.

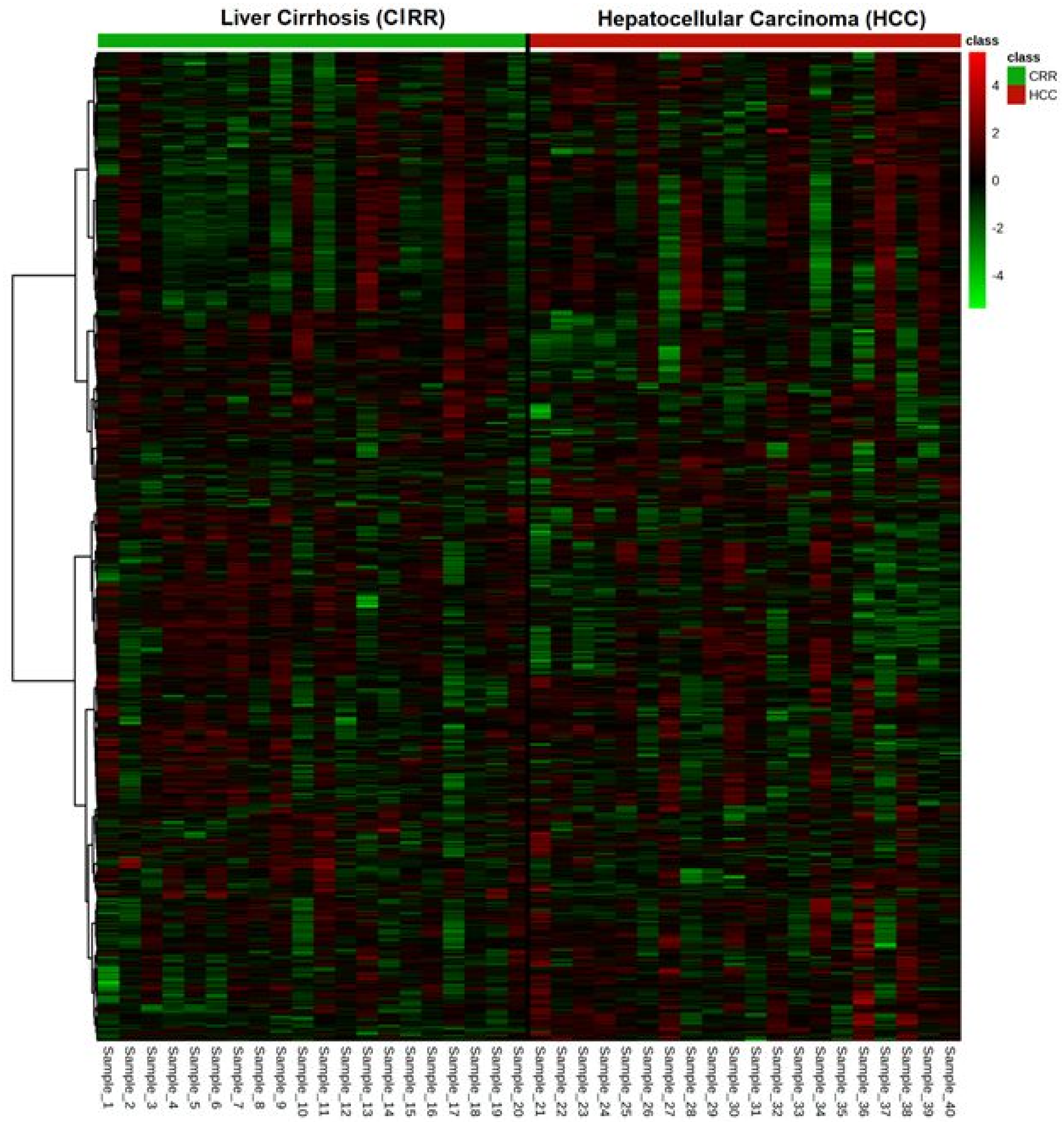

Figure S5: Heatmap of all serum endogenous peptides in HCC vs. CIRR.

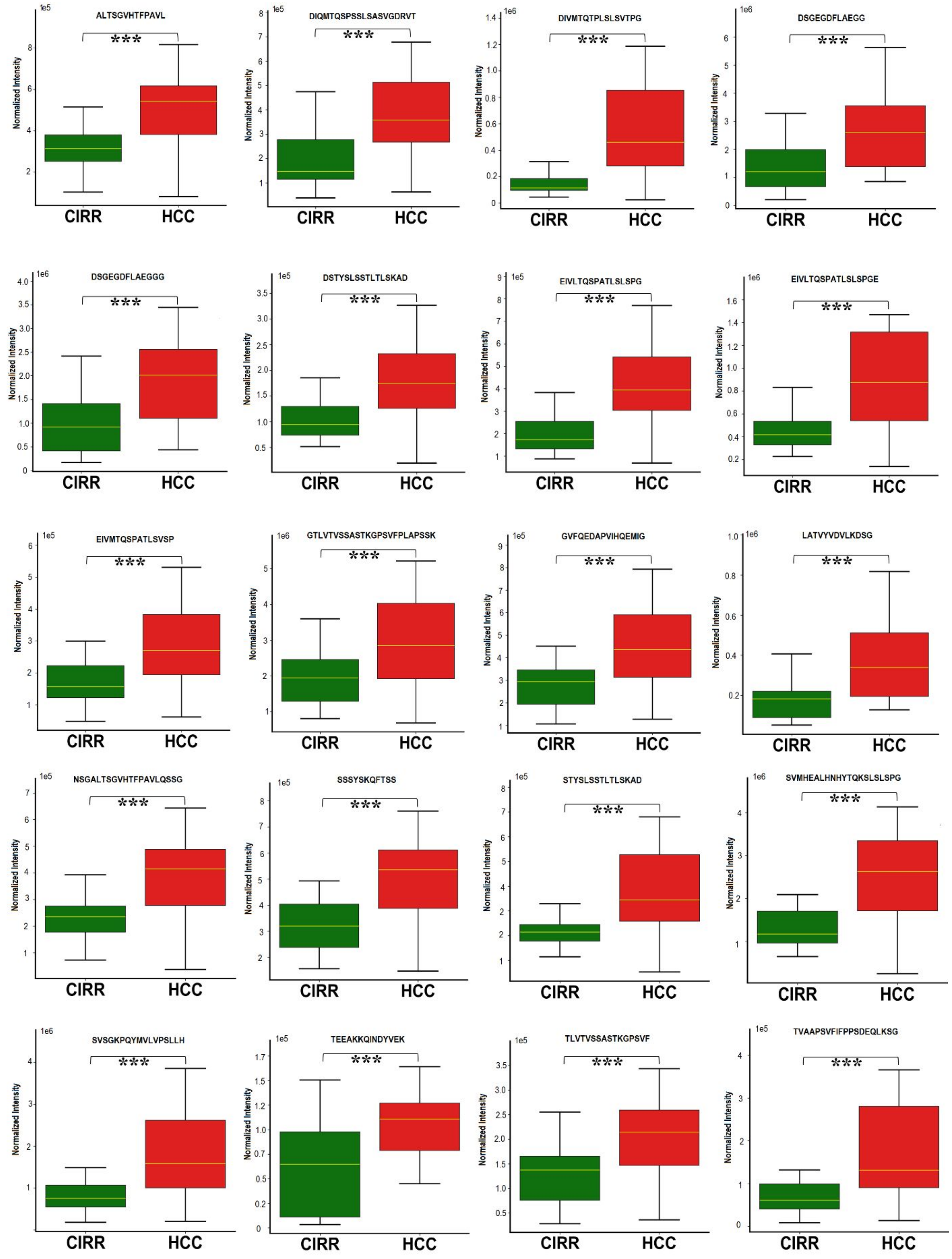

Figure S6: Box plots of significantly up-regulated serum endogenous peptides in HCC vs. CIRR. Statistical significance was denoted by * (P < 0.01), ** (P < 0.005), and *** (P < 0.0001).

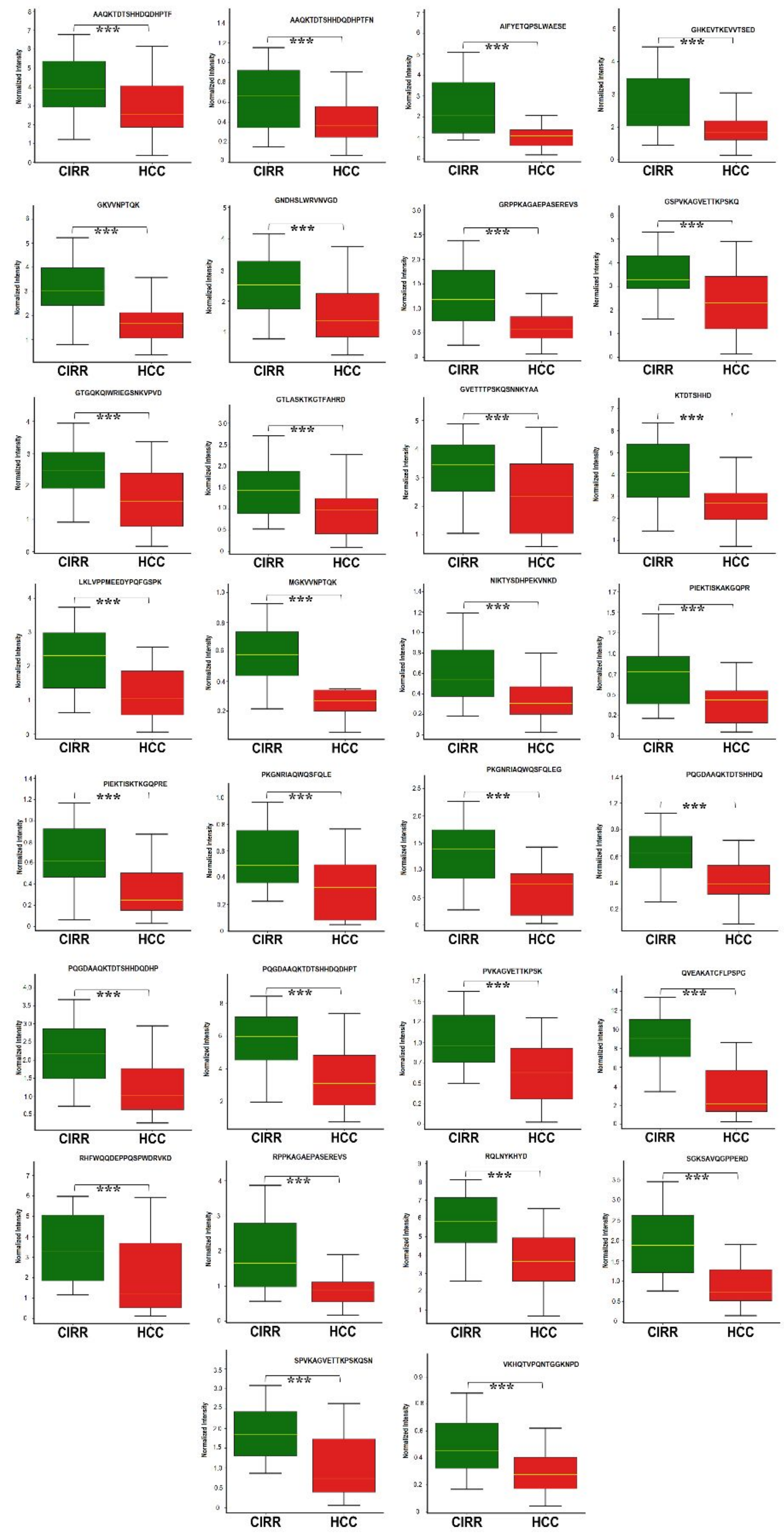

Figure S7: Box plots of significantly down-regulated serum endogenous peptides. Statistical significance was denoted by * (P < 0.01), ** (P < 0.005), and *** (P < 0.0001).

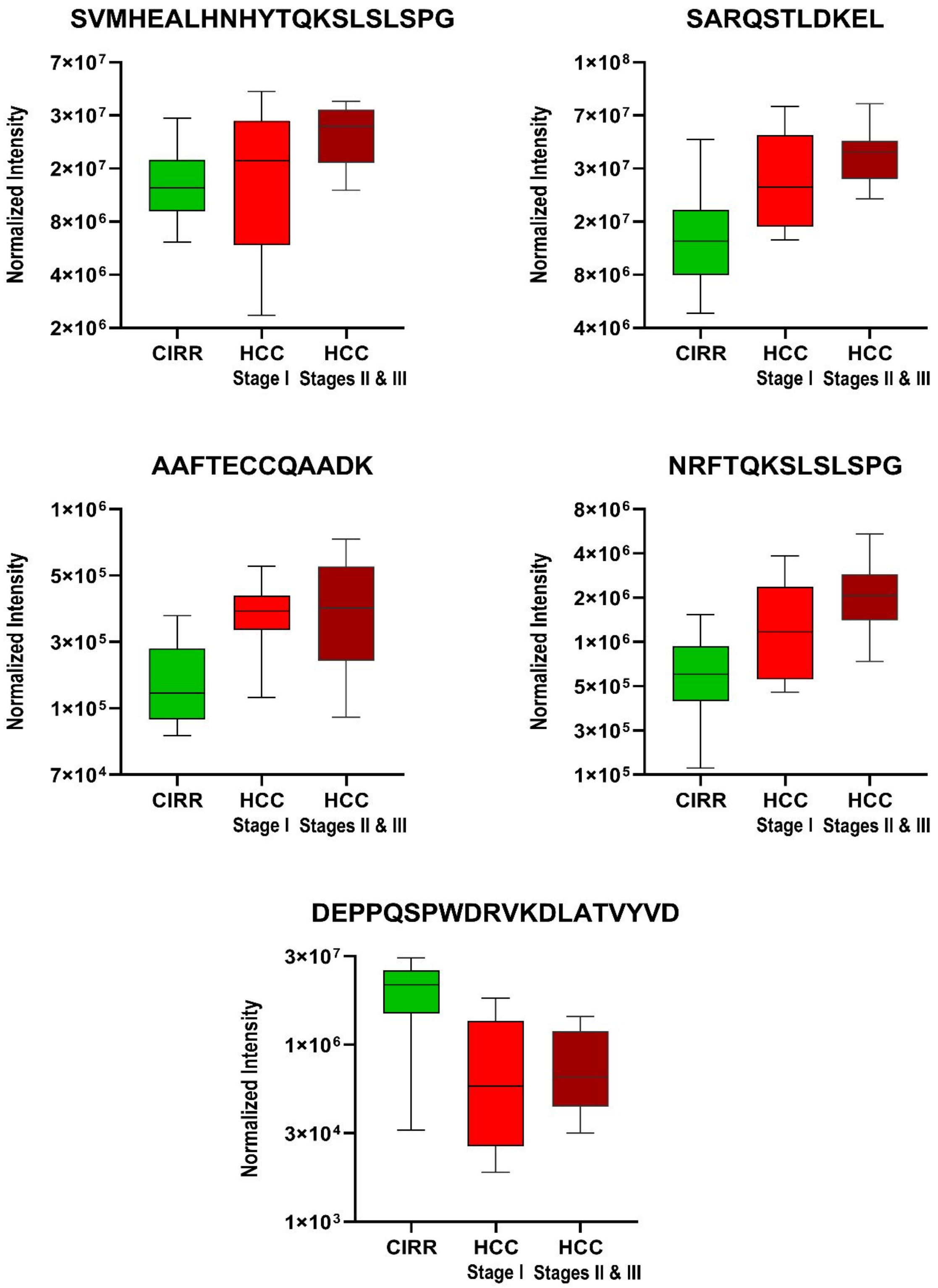

Figure S8: Comparison of candidate expressions in HCC stages I, II, III, and CIRR.

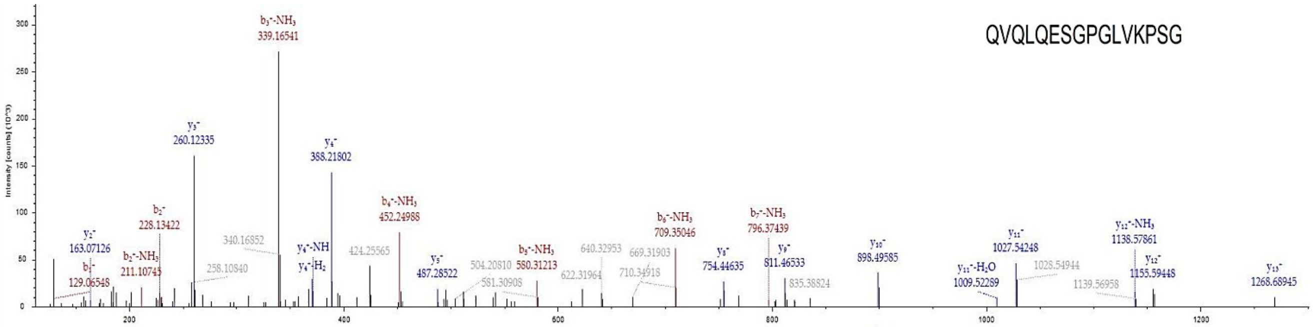
QVQLQESGPGLVKPSG

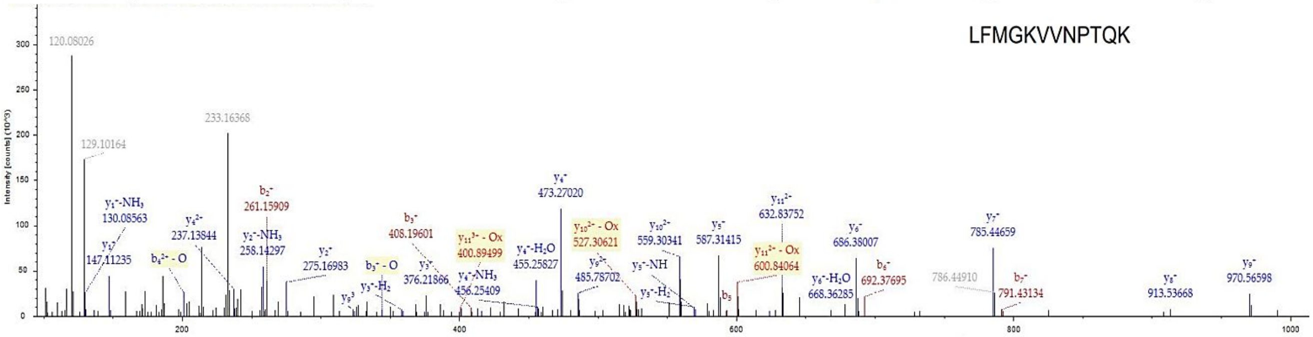
LFMGKVVNPTQK

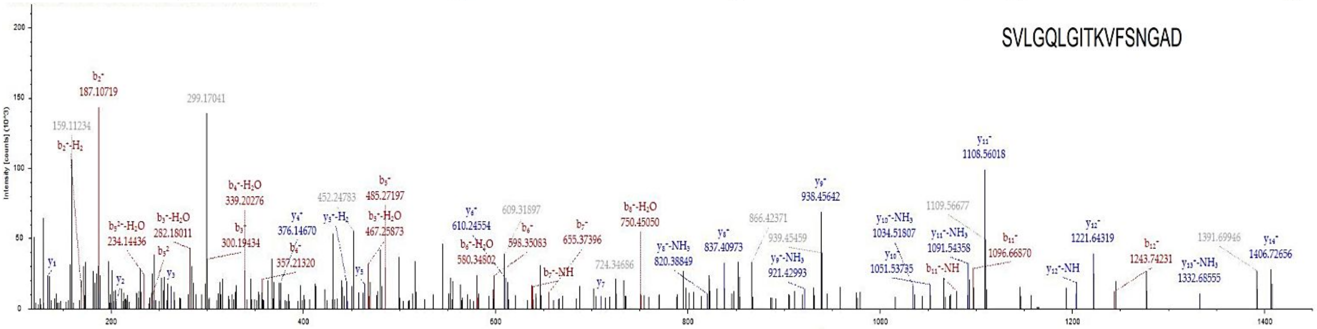
SVLGQLGITKVFSNGAD

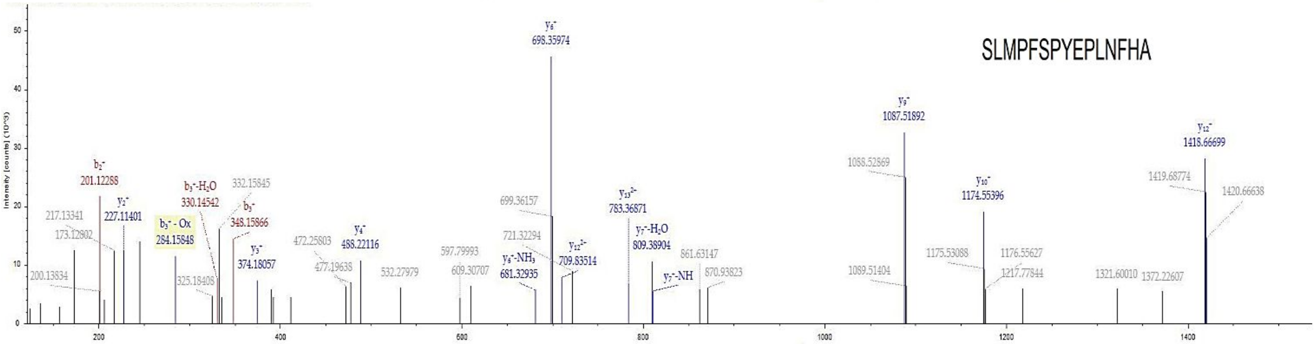
SLMPFSPYEPLNFHA

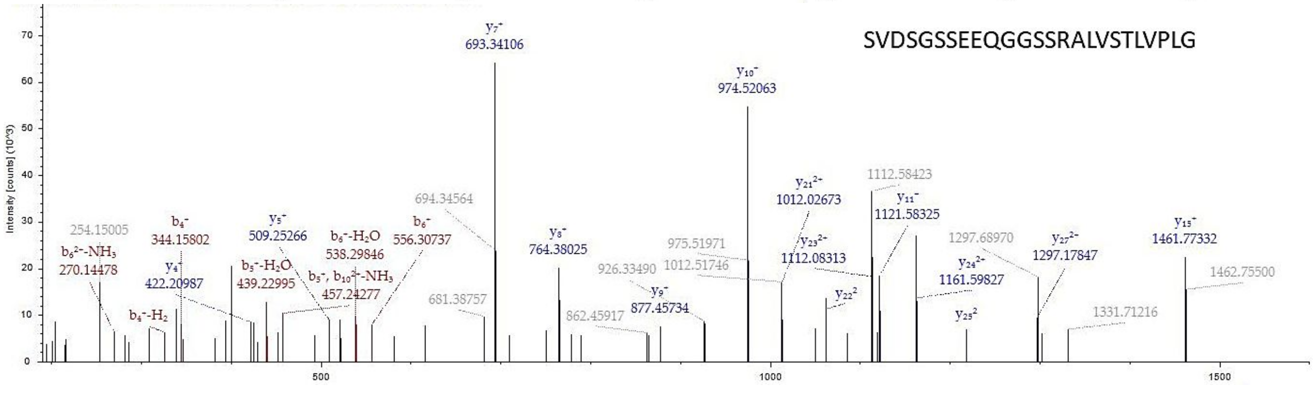
SVDSGSSEEQGGSSRALVSTLVPLG

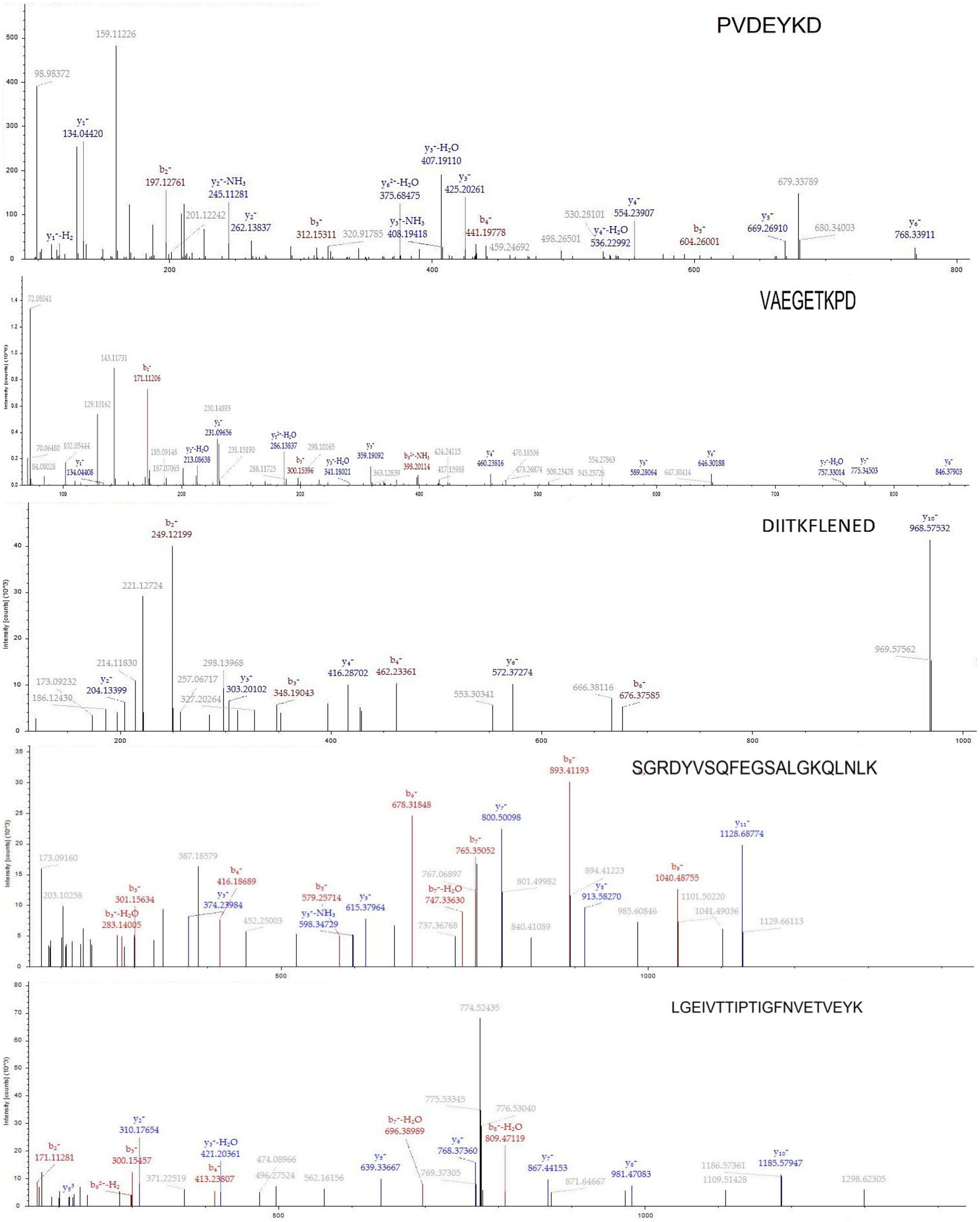

Figure S9: MS/MS spectra of peptides found in HCC only.

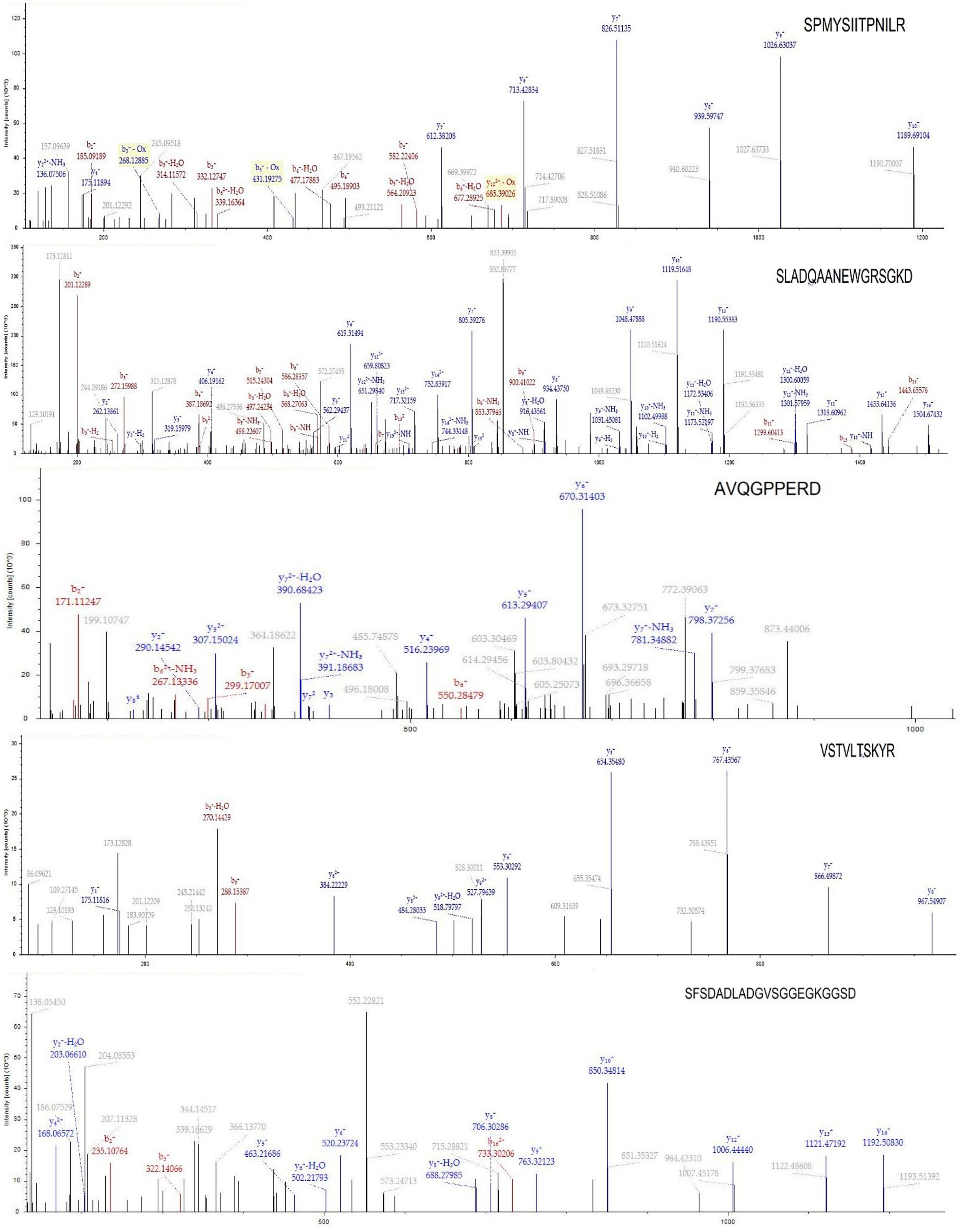
SPMYSIITPNILR
SLADQAANEWGRSGKD
AVQGPPERD
VSTVLTSKYR
SFSDADLADGVSGGEGKGGSD

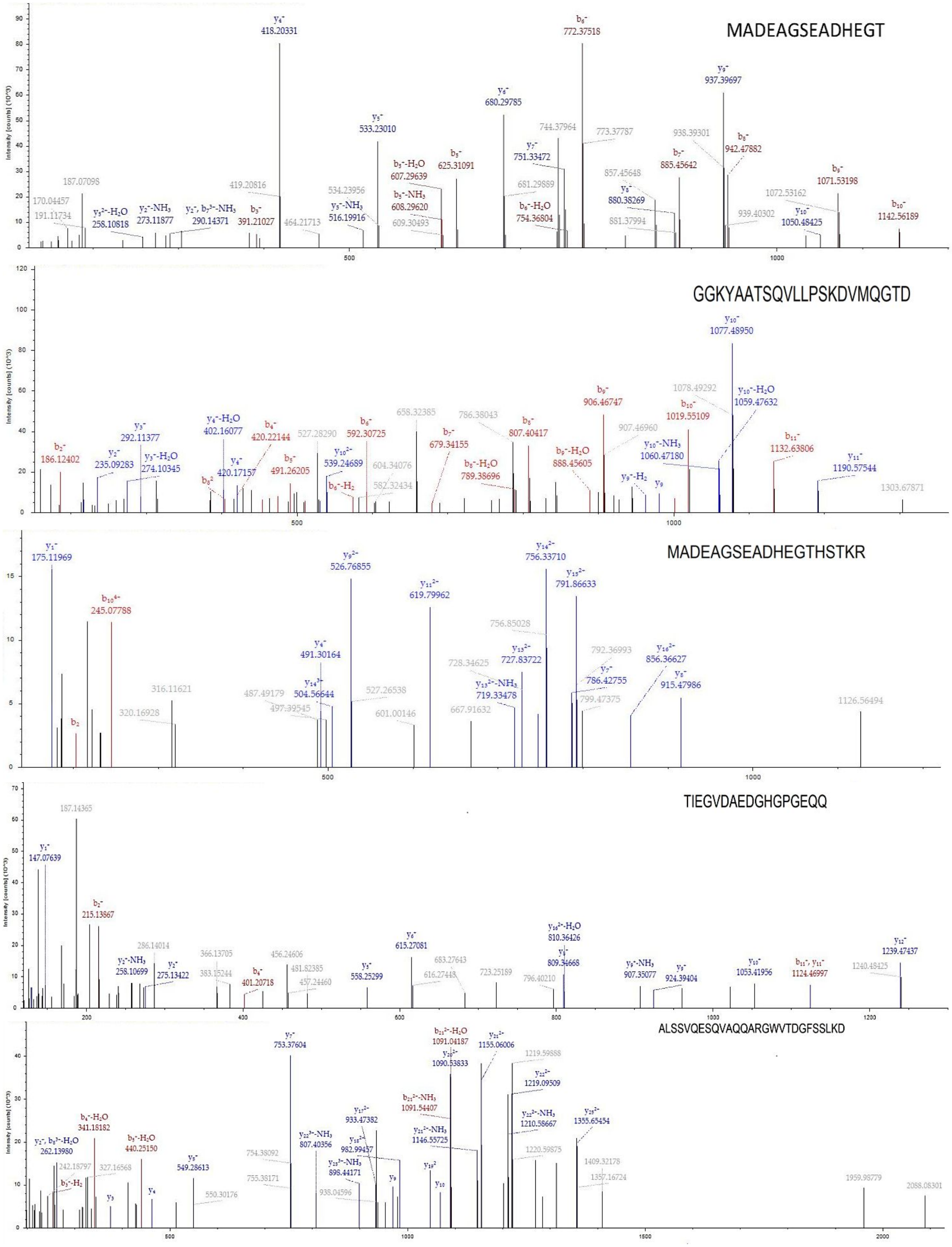

Figure S10: MS/MS spectra of peptides found in CIRR only.

Table S4: Details of endogenous peptides present over 75% in one group and completely absent in another group.

Protein Accession no	***Peptide Sequence***	***HCC***	***CIRR***
P01861	IAVEWESNGQPENNYKT	20	0
P01009	LFMGKVVNPTQK	19	0
P0DOX8	PVKAGVETTKPSKQSNN	17	0
P0DOX5	KALPAPIEKTISKAK	16	0
P00734	GLDEDSDRAIEGRTATSEYQTFFNPR	16	0
A0A075B6R2	QVQLQESGPGLVKPSG	15	0
P02656	SEAEDASLLSFMQGY	15	0
P80748	RPSGIPERFSGSNSGNTATLTISRVEAGDEAD	0	19
P01009	LFMGKVVNPTQK	0	18
P01871	GGKYAATSQVLLPSKDVMQGTD	0	17
Q15848	ADNDNDSTFTGFLLYHDTN	0	16
P01024	SPMYSIITPNILR	0	15
P01876	AVQGPPERD	0	14
P10909	SLMPFSPYEPLNFHA	0	14

Table S5: Top fifteen upstream regulators.

Upstream Regulator	***Molecule Type***	***p-value of overlap***	***Target Molecules in Dataset***
HNF4A	transcription regulator	5.38E-07	A1BG,ALB,APOA1,APOB,C2,C3,CP,FGA,GSN,KNG1,SERPINA1,TF
CEBPB	transcription regulator	5.79E-09	ALB,APOB,C3,CP,IGKC,KRT18,SERPINA1,TF
HNF1A	transcription regulator	7.45E-07	ALB,APOB,C2,C4BPA,FGA,KNG1,SERPINA1
TP53	transcription regulator	0.00376	A2M,ALB,APOA1,C2,CP,GSN,KRT18
FOXA2	transcription regulator	1.4E-07	A2M,ALB,APOA1,APOB,C3,SERPINA1,TF
STAT3	transcription regulator	0.000117	A2M,C3,CP,FGA,SERPINA1,TNS4
SMARCA4	transcription regulator	4.79E-05	A2M,ALB,APOA1,CP,KRT18,TF
SOX2	transcription regulator	0.000157	ALB,GSN,KRT18,SERPINA1,TF
NFKBIA	transcription regulator	8.59E-05	A2M,APOA1,C3,CP,KRT18
ARID1A	transcription regulator	0.00149	C3,GSN,KRT18,SERPINA1
PPARG	ligand-dependent nuclear receptor	0.00168	APOA1,C3,KRT18,SERPINA1
NR1H4	ligand-dependent nuclear receptor	0.000109	APOA1,C3,KNG1,SERPINA1

PPARA	ligand-dependent nuclear receptor	0.00131	APOA1,C2,C3,FGA
POU5F1	transcription regulator	0.000672	ALB,KRT18,SERPINA1,TF
ZFHX3	transcription regulator	2.53E-06	ALB,APOA1,KRT18,SERPINA1
SMAD3	transcription regulator	0.000366	ALB,APOA1,APOB,TF
